# What is global health? Key concepts and clarification of misperceptions: Report of the 2019 GHRP editorial meeting

**DOI:** 10.1186/s41256-020-00142-7

**Published:** 2020-04-07

**Authors:** Xinguang Chen, Hao Li, Don Eliseo Lucero-Prisno, Abu S. Abdullah, Jiayan Huang, Charlotte Laurence, Xiaohui Liang, Zhenyu Ma, Zongfu Mao, Ran Ren, Shaolong Wu, Nan Wang, Peigang Wang, Tingting Wang, Hong Yan, Yuliang Zou

**Affiliations:** 1 Global Health Institute, Wuhan University, Wuhan, China; 2 Department of Epidemiology, University of Florida, Florida, USA; 3 School of Health Sciences, Wuhan University, Wuhan, China; 4 Department of Global Health and Development, London School of Hygiene and Tropical Medicine, London, UK; 5 Global Health Research Center, Duke Kunshan University, Kunshan, China; 6 Duke Global Health Institute, Duke University, Durham, North Carolina USA; 7 School of Public Health, Fudan University, Shanghai, China; 8 Consultant in Global Health, London, UK; 9 School of Public Health, Guangxi Medical University, Guangxi, China; 10 Global Health Research Center, Dalian Medical University, Dalian, China; 11 School of Public Health, Sun Yat-sen University, Guangzhou, China

**Keywords:** Global health, Definition, Global impact, Global solution, Global health sciences, Global health theory, global health education, Global health development

## Abstract

The call for “W*orking Together to Build a Community of Shared Future for Mankind”* requires us to improve people’s health across the globe, while global health development entails a satisfactory answer to a fundamental question: “What is global health?” To promote research, teaching, policymaking, and practice in global health, we summarize the main points on the definition of global health from the Editorial Board Meeting of Global Health Research and Policy, convened in July 2019 in Wuhan, China. The meeting functioned as a platform for free brainstorming, in-depth discussion, and post-meeting synthesizing. Through the meeting, we have reached a consensus that global health can be considered as a general guiding principle, an organizing framework for thinking and action, a new branch of sciences and specialized discipline in the large family of public health and medicine. The word “global” in global health can be subjective or objective, depending on the context and setting. In addition to dual-, multi-country and global, a project or a study conducted at a local area can be global if it (1) is framed with a global perspective, (2) intends to address an issue with global impact, and/or (3) seeks global solutions to an issue, such as frameworks, strategies, policies, laws, and regulations. In this regard, global health is eventually an extension of “international health” by borrowing related knowledge, theories, technologies and methodologies from public health and medicine. Although global health is a concept that will continue to evolve, our conceptualization through group effort provides, to date, a comprehensive understanding. This report helps to inform individuals in the global health community to advance global health science and practice, and recommend to take advantage of *the Belt and Road Initiative* proposed by China.

“Promoting *Health For All”* can be considered as the mission of global health for collective efforts to build “a *Community of Shared Future for Mankind”* first proposed by President Xi Jinping of China in 2013. The concept of global health continues to evolve along with the rapid development in global health research, education, policymaking, and practice. It has been promoted on various platforms for exchange, including conferences, workshops and academic journals. Within the Editorial Board of Global Health Research and Policy (GHRP), many members expressed their own points of view and often disagreed with each other with regard to the concept of global health. Substantial discrepancies in the definition of global health will not only affect the daily work of the Editorial Board of GHRP, but also impede the development of global health sciences.

To promote a better understanding of the term “*global health”*, we convened a special session in the 2019 GHRP Editorial Board Meeting on the 7th of July at Wuhan University, China. The session started with a review of previous work on the concept of global health by researchers from different institutions across the globe, followed by free brainstorms, questions-answers and open discussion. Individual participants raised many questions and generously shared their thoughts and understanding of the term global health. The session was ended with a summary co-led by Dr. Xinguang Chen and Dr. Hao Li. Post-meeting efforts were thus organized to further synthesize the opinions and comments gathered during the meeting and post-meeting development through emails, telephone calls and in-person communications. With all these efforts together, concensus have been met on several key concepts and a number of confusions have been clarified regarding global health. In this editorial, we report the main results and conclusions.

## A brief history

Our current understanding of the concept of global health is based on information in the literature in the past seven to eight decades. Global health as a scientific term first appeared in the literature in the 1940s [1]. It was subsequently used by the World Health Organization (WHO) as guidance and theoretical foundation [2–4]. Few scholars discussed the concept of global health until the 1990s, and the number of papers on this topic has risen rapidly in the subsequent decade [5] when global health was promoted under *the Global Health Initiative* - a global health plan signed by the U.S. President Barack Obama [6]. As a key part of the national strategy in economic globalization, security and international policies, global health in the United States has promoted collaborations across countries to deal with challenging medical and health issues through federal funding, development aids, capacity building, education, scientific research, policymaking and implementation.

Based on his experience working with Professor Zongfu Mao, the lead Editors-in-Chief, who established the Global Health Institute at Wuhan University in 2011 and launched the GHRP in 2016, Dr. Chen presented his own thoughts surrounding the definition of global health to the 2019 GHRP Editorial Board Meeting. Briefly, Dr. Chen defined global health with a three-dimensional perspective.

First, global health can be considered as a guiding principle, a branch of health sciences, and a specialized discipline within the broader arena of public health and medicine [5]. As many researchers posit, global health first serves as a guiding principle for people who would like to contribute to the health of all people across the globe [5, 7, 8].

Second, Dr. Chen’s conceptualization of global health is consistent with the opinions of many other scholars. Global health as a branch of sciences focuses primarily on the medical and health issues with global impact or can be effectively addressed through global solutions [9–16]. Therefore, the goal of global health science is to understand global medical and health issues and develop global solutions and implications [7, 9, 15, 17–19].

Third, according to Dr. Chen, to develop global health as a branch of science in the fields of public health and medicine, a specialized discipline must be established, including educational institutions, research entities, and academic societies. Only with such infrastructure, can the professionals and students in the global health field receive academic training, conduct global health research, exchange and disseminate research findings, and promote global health practices [5, 15, 20–23].

Developmentally and historically, we have learned and will continue to learn global health from the WHO [1, 4, 24, 25]. WHO’s projects are often ambitious, involving multiple countries, or even global in scope. Through research and action projects, the WHO has established a solid knowledge base, relevant theories, models, methodologies, valuable data, and lots of experiences that can be directly used in developing global health [26–29]. Typical examples include WHO’s efforts for global HIV/AIDS control [13, 30–32], and the Primary Healthcare Programs to promote Health For All [33, 34].

## The definition of Global Health

From published studies in the international literature and our experiences in research, training, teaching and practice, our meeting reached a consensus-global health is a newly established branch of health sciences, growing out from medicine, public health and international health, with much input from the WHO. What makes global health different from them is that (1) global health deals with only medical and health issues with *global impact* [35, 5, 36, 10, 14, 2] the main task of global health is to seek for global solutions to the issues with global health impact [7, 18, 37]; and (3) the ultimate goal is to use the power of academic research and science to promote health for all, and to improve health equity and reduce health disparities [7, 14, 15, 18, 38]. Therefore, global health targets populations in all countries and involves all sectors beyond medical and health systems, although global health research and practice can be conducted locally [39].

As a branch of medical and health sciences, global health has three fundamental tasks: (1) *to master* the spatio-temporal patterns of a medical and/or health issue across the globe to gain a better understanding of the issue and to assess its global impact [40–43]; (2) *to investigate* the determinants and influential factors associated with medical and health issues that are known to have global impact [15, 40–43]; and (3) *to establish* evidence-based global solutions, including strategies, frameworks, governances, policies, regulations and laws [14, 15, 28, 38, 44–47].

Like public health, medicine, and other branches of sciences, global health should have *three basic functions*: The first function is to generate new knowledge and theories about global health issues, influential factors, and develop global solutions. The second function is to distribute the knowledge through education, training, publication and other forms of knowledge sharing. The last function is to apply the global health knowledge, theories, and intervention strategies in practice to solve global health problems.

## Understanding the word “global”

Confusion in understanding the term ‘global health’ has largely resulted from our understanding of the word “global”. There are few discrepancies when the word ‘global’ is used in other settings such as in geography. In there, the world global physically pertains to the Earth we live on, including all people and all countries in the world. However, discrepancies appear when the word “global” is combined with the word “health” to form the term “global health”. Following the word “global” literately, an institution, a research project, or an article can be considered as global *only if* it encompasses all people and all countries in the world. If we follow this understanding, few of the work we are doing now belong to global health; even the work by WHO are for member countries only, not for all people and all countries in the world. But most studies published in various global health journals, including those in our GHRP, are conducted at a local or international level. How could this global health happen?

The argument presented above leads to another conceptualization: Global health means health for a very large group of people in a very large geographic area such as the Western Pacific, Africa, Asia, Europe, and Latin America. Along with this line of understanding, an institution, a research project or an article involving multi-countries and places can be considered as global, including those conducted in countries involved in China’s *Belt and Road Initiative* (BRI) [26, 48–51]. They are considered as global because they meet our definitions of global health which focus on medical and health issues with global impact or look for global solutions to a medical or health issue [5, 7, 22].

One step further, the word ‘global’ can be considered as a concept of goal-setting in global health. Typical examples of this understanding are the goals established for a global health institution, for faculty specialized in global health, and for students who major or minor in global health. Although few of the global health institutions, scholars and students have conducted or are going to conduct research studies with a global sample or delivered interventions to all people in all countries, all of them share a common goal: Preventing diseases and promoting health for all people in the world. For example, preventing HIV transmission within Wuhan would not necessarily be a global health project; but the same project can be considered as global if it is guided by a global perspective, analyzed with methods with global link such as phylogenetic analysis [52, 53], and the goal is to contribute to global implications to end HIV/AIDS epidemic.

## The concept of global impact

Global impact is a key concept for global health. Different from other public health and medical disciplines, global health can address any issue that has a global impact on the health of human kind, including health system problems that have already affected or will affect a large number of people or countries across the globe. Three illustrative examples are (1) the SARS epidemic that occurred in several areas in Hong Kong could spread globally in a short period [11] to cause many medical and public health challenges [54, 55]; (2) the global epidemic of HIV/AIDS [13]; and the novel coronavirus epidemic first broke out in December 2019 in Wuhan and quickly spread to many countries in the world [56].

Along with rapid and unevenly paced globalization, economic growth, and technological development, more and more medical and health issues with global impact emerge. Typical examples include growing health disparities, migration-related medical and health issues, issues related to internet abuse, the spread of sedentary lifestyles and lack of physical activity, obesity, increasing rates of substance abuse, depression, suicide and many other emerging mental health issues, and so on [10, 23, 36, 42, 57–60]. GHRP is expecting to receive and publish more studies targeting these issues guided by a global health perspective and supports more researchers to look for global solutions to these issues.

## The concept of global solution

Another concept parallel to global impact is *global solution*. What do we mean by global solutions? Different from the conventional understanding in public health and medicine, global health selectively targets issues with global impact. Such issues often can *only* be effectively solved at the macro level through cross-cultural, international, and/or even global collaboration and cooperation among different entities and stakeholders. Furthermore, as long as the problem is solved, it will benefit a large number of population. We term this type of interventions as a global solution. For example, the 90–90-90 strategy promoted by the WHO is a global solution to end the HIV/AIDS epidemic [61, 62]; the measures used to end the SARS epidemic is a global solution [11]; and the ongoing measures to control influenza [63, 64] and malaria [45, 65], and the measures taken by China, WHO and many countries in the world to control the new coronaviral epidemic started in China are also great examples of global solutions [66].

Global solutions are also needed for many emerging health problems, including cardiovascular diseases, sedentary lifestyle, obesity, internet abuse, drug abuse, tobacco smoking, suicide, and other problems [29, 44]. As described earlier, global solutions are not often a medical intervention or a procedure for individual patients but frameworks, policies, strategies, laws and regulations. Using social media to deliver interventions represents a promising approach in establishment of global solutions, given its power to penetrate physical barriers and can reach a large body of audience quickly.

## Types of Global Health researches

One challenge to GHRP editors (and authors alike) is how to judge whether a research study is global? Based on the new definition of global health we proposed as described above, two types of studies are considered as global and will receive further reviews for publication consideration. Type I includes projects or studies that involve multiple countries with diverse backgrounds or cover a large diverse populations residing in a broad geographical area. Type II includes projects or studies guided by a global perspective, although they may use data from a local population or a local territory. Relative to Type I, we anticipate more Type II project and studies in the field of global health. Type I study is easy to assess, but caution is needed to assess if a project or a study is Type II. Therefore, we propose the following three points for consideration: (1) if the targeted issues are of global health impact, (2) if the research is attempted to understand an issue with a global perspective, and (3) if the research purpose is to seek for a global solution.

An illustrative example of Type I studies is the epidemic and control of SARS in Hong Kong [11, 67]. Although started locally, SARS presents a global threat; while controlling the epidemic requires international and global collaboration, including measures to confine the infected and measures to block the transmission paths and measures to protect vulnerable populations, not simply the provisions of vaccines and medicines. HIV/AIDS presents another example of Type I project. The impact of HIV/AIDS is global. Any HIV/AIDS studies regardless of their scope will be global as long as it contributes to the global efforts to end the HIV/AIDS epidemic by 2030 [61, 62]. Lastly, an investigation of cardiovascular diseases (CVD) in a country, in Nepal for example, can be considered as global if the study is framed from a global perspective [44].

The discussion presented above suggests that in addition to scope, *the purpose* of a project or study can determine if it is global. A pharmaceutical company can target all people in the world to develop a new drug. The research would be considered as global if the purpose is to improve the medical and health conditions of the global population. However, it would not be considered as global if the purpose is purely to pursue profit. A research study on a medical or health problem among rural-to-urban migrants in China [57, 58, 60] can be considered as global if the researchers frame the study with a global perspective and include an objective to inform other countries in the world to deal with the same or similar issues.

## Think globally and act locally

The catchphrase “think globally and act locally” presents another guiding principle for global health and can be used to help determine whether a medical or public health research project or a study is global. First, thinking globally and acting locally means to learn from each other in understanding and solving local health problems with the broadest perspective possible. Taking traffic accidents as an example, traffic accidents increase rapidly in many countries undergoing rapid economic growth [68, 69]. There are two approaches to the problem: (1) locally focused approach: conducting research studies locally to identify influential factors and to seek for solutions based on local research findings; or (2) a globally focused approach: conducting the same research with a global perspective by learning from other countries with successful solutions to issues related traffic accidents [70].

Second, thinking globally and acting locally means adopting solutions that haven been proven effective in other comparable settings. It may greatly increase the efficiency to solve many global health issues if we approach these issues with a globally focused perspective. For example, vector-borne diseases are very prevalent among people living in many countries in Africa and Latin America, such as malaria, dengue, and chikungunya [45, 71, 72]. We would be able to control these epidemics by directly adopting the successful strategy of massive use of bed nets that has been proven to be effective and cost-saving [73]. Unfortunately, this strategy is included only as “simple alternative measures” in the so-called global vector-borne disease control in these countries, while most resources are channeled towards more advanced technologies and vaccinations [16, 19, 74].

Third, thinking globally and acting locally means learning from each other at different levels. At the individual level, people in high income countries can learn from those in low- and mid-income countries (LMICs) to be physically more active, such as playing Taiji, Yoga, etc.; while people in LMICs can learn from those in high income countries to improve their hygiene, life styles, personal health management, etc. At the population level, communities, organizations, governments, and countries can learn from each other in understanding their own medical and health problems and healthcare systems, and to seek solutions for these problems. For example, China can learn from the United States to deal with health issues of rural to urban migrants [75]; and the United States can learn from China to build three-tier health care systems to deliver primary care and prevention measures to improve health equality.

Lastly, thinking globally and acting locally means opportunities to conduct global health research and to be able to exchange research findings and experiences across the globe; even without traveling to another country. For example, international immigrants and international students present a unique opportunity for global health research in a local city [5, 76]. To be global, literature search and review remains the most important approach for us to learn from each other besides conducting collaborative work with the like-minded researchers across countries; rapid development in big data and machine learning provide another powerful approach for global health research. Institutions and programs for global health provides a formal venue for such learning and exchange opportunities.

## Reframing a local research study as global

The purpose of this article is to promote global health through research and publication. Anyone who reads this paper up to this point might already be able to have a clear idea on how to reframe his/her own research project or article to be of global nature. There is no doubt that a research project is global if it involves multiple countries with investigators of diverse backgrounds from different countries. However, if a research project targets a local population with investigators from only one or two local institutions, can such project be considered as global?

Our answer to this question is “yes” even if a research study is conducted locally, if the researcher (1) can demonstrate that the issue to be studied or being studied has a global impact, or (2) eventually looks for a global solution although supported with local data. For example, the study of increased traffic accidents in a city in Pakistan can be considered as global if the researchers frame the problem from a global perspective and/or adopt global solutions by learning from other countries. On the other hand, a statistical report of traffic accidents or an epidemiological investigation of factors related to the traffic accidents at the local level will not be considered as global. Studies conducted in a local hospital on drug resistance to antibiotics and associated cost are global if expected findings can inform other countries to prevent abuse of antibiotics [77]. Lastly, studies supported by international health programs can be packaged as global simply by broadening the vision from international to global.

## Is Global Health a new bottle with old wine?

Another challenge question many scholars often ask is: “What new things can global health bring to public health and medicine?” The essence of this question is whether global health is simply a collection of existing medical and health problems packaged with a new title? From our previous discussion, many readers may already have their own answer to this question that this is not true. However, we would like to emphasize a few points. First, global health is not equal to public health, medicine or both, but a newly emerged sub-discipline within the public health-medicine arena. Global health is not for all medical and health problems but for the *problems with global impact* and with the purpose of seeking global solutions. In other words, global health focuses primarily on mega medical and health problems that transcend geographical, cultural, and national boundaries and seeks broad solutions, including frameworks, partnerships and cooperation, policies, laws and regulations that can be implemented through governments, social media, communities, and other large and broad reaching mechanisms.

Second, global health needs many visions, methods, strategies, approaches, and frameworks that are not conventionally used in public health and medicine [5, 18, 22, 34]. They will enable global health researchers to locate and investigate those medical and health issues with global impact, gain new knowledge about them, develop new strategies to solve them, and train health workers to deliver the developed strategies. Consequently, geography, history, culture, sociology, governance, and laws that are optional for medicine and public health are essential for global health. Lastly, it is fundamental to have a global perspective for anyone in global health, but this could be optional for other medical and health scientists [40, 41].

## Global Health, international health, and public health

As previously discussed, global health has been linked to several other related disciplines, particularly public health, international health, and medicine [3, 5, 7, 18, 22]. To our understanding, global health can be considered as an application of medical and public health sciences together with other disciplines (1) in tackling those issues with global impact and (2) in the effort to seek global solutions. Thus, global health treats public health sciences and medicine as their foundations, and will selectively use theories, knowledge, techniques, therapeutics and prevention measures from public health, medicine, and other disciplines to understand and solve global health problems.

There are also clear boundaries between global health, public health and medicine with regard to the target population. Medicine targets patient populations, public health targets health populations in general, while global health targets the global population. We have to admit that there are obvious overlaps between global health, public health and medicine, particularly between global health and international health. It is worth noting that global health can be considered as an extension of international health with regard to the scope and purposes. International health focuses on the health of participating countries with intention to affect non-participating countries, while global health directly states that its goal is to promote health and prevent and treat diseases for all people in all countries across the globe. Thus, global health can be considered as developed from, and eventually replace international health.

## Challenges and opportunities for China to contribute to Global Health

To pursue *A Community with a Shared Future for Mankind*, China’s *BRI*, currently involving more than 150 countries across the globe, creates a great opportunity for Chinese scholars to contribute to global health. China has a lot to learn from other countries in advancing its medical and health technologies and to optimize its own healthcare system, and to reduce health disparities among the 56 ethnic groups of its people. China can also gain knowledge from other countries to construct healthy lifestyles and avoid unhealthy behaviors as Chinese people become more affluent. Adequate materials and money may be able to promote physical health in China; but it will be challenging for Chinese people to avoid mental health problems currently highly prevalent in many rich and developed countries.

To develop global health, we cannot ignore the opportunities along with the BRI for Chinese scholars to share China’s lessons and successful experience with other countries. China has made a lot of achievements in public health and medicine before and after the Open Door Policy [49, 78]. Typical examples include the ups and downs of the 3-Tier Healthcare Systems, the Policy of Prevention First, and the Policy of Putting Rural Health as the Priority, the Massive Patriotic Hygiene Movement with emphasis on simple technology and broad community participation, the Free Healthcare System for urban and the Cooperative Healthcare System for rural residents. There are many aspects of these initiatives that other countries can emulate including the implementation of public health programs covering a huge population base unprecedented in many other countries.

There are challenges for Chinese scholars to share China’s experiences with others as encountered in practice. First of all, China is politically very stable while many other countries have to change their national leadership periodically. Changes in leadership may result in changes in the delivery of evidence- based intervention programs/projects, although the changes may not be evidence-based but politically oriented. For example, the 3-Tier Healthcare System that worked in China [79, 80] may not work in other countries and places without modifications to suit for the settings where there is a lack of local organizational systems. Culturally, promotion of common values among the public is unique in China, thus interventions that are effective among Chinese population may not work in countries and places where individualism dominates. For example, vaccination program as a global solution against infectious diseases showed great success in China, but not in the United States as indicated by the 2019 measles outbreak [81].

China can also learn from countries and international agencies such as the United Kingdom, the United States, the World Health Organization, and the United Nations to successfully and effectively provide assistance to LMICs. As China develops, it will increasingly take on the role of a donor country. Therefore, it is important for Chinese scholars to learn from all countries in the world and to work together for a Community of Shared Future for Mankind during the great course to develop global health.

## Conclusion

Promotion of global health is an essential part of the Working Together  to Build a Community of Shared Future for Mankind. In this editorial, we summarized our discussions in the 2019 GHRP Editorial Board Meeting regarding the concept of global health. The goal is to enhance consensus among the board members as well as researchers, practitioners, educators and students in the global health community. We welcome comments, suggestions and critiques that may help further our understanding of the concept. We would like to keep the concept of global health open and let it evolve along with our research, teaching, policy and practice in global health.
